# Efficacy of left atrial plication for atrial functional mitral regurgitation

**DOI:** 10.1007/s11748-020-01483-3

**Published:** 2020-09-20

**Authors:** Masamichi Matsumori, Motoharu Kawashima, Takamitsu Aihara, Jun Fujisue, Masato Fujimoto, Keigo Fukase, Yoshikatsu Nomura, Hiroshi Tanaka, Hirohisa Murakami, Nobuhiko Mukohara

**Affiliations:** Department of Cardiovascular Surgery, Hyogo Brain and Heart Center At Himeji, 520 Saisho-Ko, Himeji, 670-0981 Japan

**Keywords:** Atrial functional mitral regurgitation, Atriogenic leaflet tethering, Giant left atrium, Left atrial plication

## Abstract

**Objective:**

Atrial functional mitral regurgitation (AFMR) is caused by atrial fibrillation and left atrial enlargement. Our study aimed to evaluate the efficacy of left atrial plication (LAP) for AFMR.

**Methods:**

Of 1164 mitral valve surgery patients at our hospital from January 2000 to May 2019, 22 patients underwent surgery for AFMR. Our retrospective analysis divided the patients with AFMR into two groups according to whether LAP was performed (LAP + group, *n* = 9; LAP − group, *n* = 13). Mitral valve angle (MV angle) (horizontal inclination of mitral valve) was measured by pre- and post-operative computed tomography scan. Individuals with type II mitral regurgitation, left ventricular ejection fraction of < 55%, males with left ventricular endo-diastolic dimension of > 60 mm and females with > 55 mm, aortic valve disease, mitral valve calcification, hypertrophic obstructive cardiomyopathy, and both “redo” and emergency cases were excluded.

**Result:**

Mitral valve replacement was performed in 6 patients and mitral ring annuloplasty in 16 cases. No recurrence of mitral regurgitation or structural valve deterioration occurred during the follow-up period. There were no hospital deaths; 3 deaths occurred during the follow-up period. Compared to the LAP − group, the LAP + group demonstrated a significantly greater decrease of MV angle (16.6 ± 8.1° vs. 1.2 ± 6.9°, *p* < 0.01) and left atrial dimension (18.4 ± 7.0 mm vs. 6.9 ± 14.6 mm, *p* = 0.02).

**Conclusions:**

Surgical results of AFMR were satisfactory. LAP may be appropriate for correcting the angle of a mitral valve tilted horizontally. More cases need to be considered in the future.

## Introduction

Functional mitral regurgitation (FMR) caused by left ventricular (LV) dysfunction or myocardial infarction has been appreciated and the therapeutic strategy well discussed over recent decades [1–3]. In contrast, FMR caused by atrial fibrillation and LA enlargement (AFMR) [4–7] has received less attention but is increasingly acknowledged. Several investigations have sought to clarify the mechanism of AFMR by two-dimensional (2D) or 3D transesophageal echocardiography (TEE) [8.9], generating increased interest. However, management of AFMR including surgical repair [10, 11] is still controversial. Our study aimed to evaluate surgical outcome and the efficacy of left atrial plication (LAP) for AFMR with giant LA.

## Patients and methods

### Patients

**Fig. 1:**
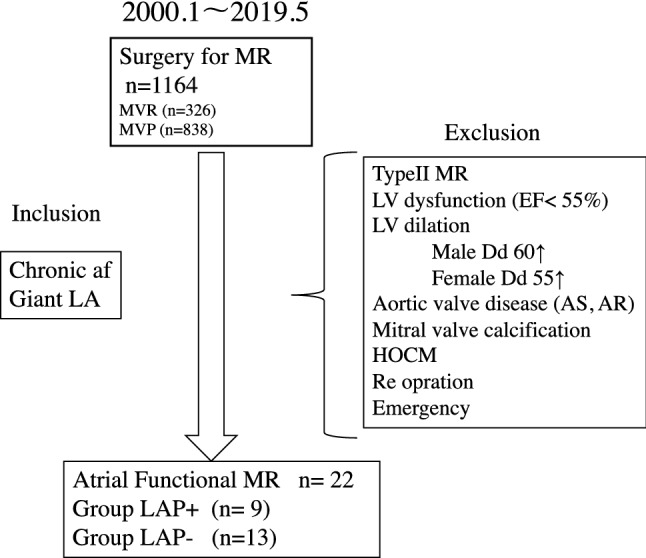
Flowchart of the study of atrial functional. MR *MR* mitral valve regurgitation, *AF* atrial fibrillation, *LA* left atrium, *LV* left ventricular, *Dd* end-diastolic dimension, *Ds* end-systolic dimension, *AS* aortic valve stenosis, *AR* aortic valve regurgitation, *HOCM* hypertrophic obstructive cardiomyopathy *LAP* left atrial plication,

Of 1164 patients who underwent mitral valve surgery at our hospital, we retrospectively studied 22 patients who MV surgery was indicated for AFMR. All patients had chronic AF ≥ 3 years duration with LA enlargement (LA diameter > 40 mm). Patients with functional MR (LV dysfunction), aortic valve disease, redo cases, and emergency cases were excluded from the analysis. We divided into two groups according to perform LAP: LAP + group consist of 9 patients who was performed LAP and LAP − group consist of 13 patients who was not performed LAP (Fig. 1). Preoperative patient characteristics are given in Table 1. Mean age was 73.5 ± 5.7 (61–83) years, and mean body surface area was 1.54 ± 0.38 (1.35–1.94) m^2^. 7 patients (31.8%) were NHYA (New York Heart Association) functional class greater than III.

**Table 1:** Preoperative patients characteristics

Variables	Total (*N* = 22)	LAP + (*N* = 9)	LAP − (*N* = 13)	*P* value (LAP + vs. LAP − )
Age (years)	73.5 ± 5.7	71.1 ± 6.8	75.2 ± 4.3	0.14
Male gender (%)	15 (68.1)	7 (77.7)	8 (61.5)	0.43
BSA (/m^2^)	1.54 ± 0.38	1.68 ± 0.20	1.45 ± 0.45	0.13
NYHA I (%)	4 (18.1)	0	4 (30.7)	0.04
II (%)	11 (47.8)	6 (66.6)	5 (38.4)	0.21
III (%)	6 (27.2)	3 (33.3)	3 (23.1)	0.63
IV (%)	1 (4.5)	0	1 (7.7)	0.34
Hypertension (%)	4 (18.1)	3 (33.3)	1 (7.7)	0.19
Hyperlipidemia (%)	6 (27.2)	1 (11.1)	5 (38.4)	0.14
COPD (%)	14 (63.6)	7 (77.7)	7 (53.8)	0.26
Diabetes (%)	6 (27.2)	4 (44.4)	2 (15.4)	0.18
Smoke (%)	8 (36.3)	4 (44.4)	4 (30.8)	0.54

*LAP* left atrial plication, *BSA* body surface area, *NYHA* New York Heart Association, *COPD* chronic obstructive pulmonary disease

Patient permission with informed consent was obtained for retrospectively analyzing and reporting these results. The report was reviewed and approved by the Institutional Review Board of Hyogo Brain and Heart Center at Himeji.

### Echocardiography

**Fig. 2:**
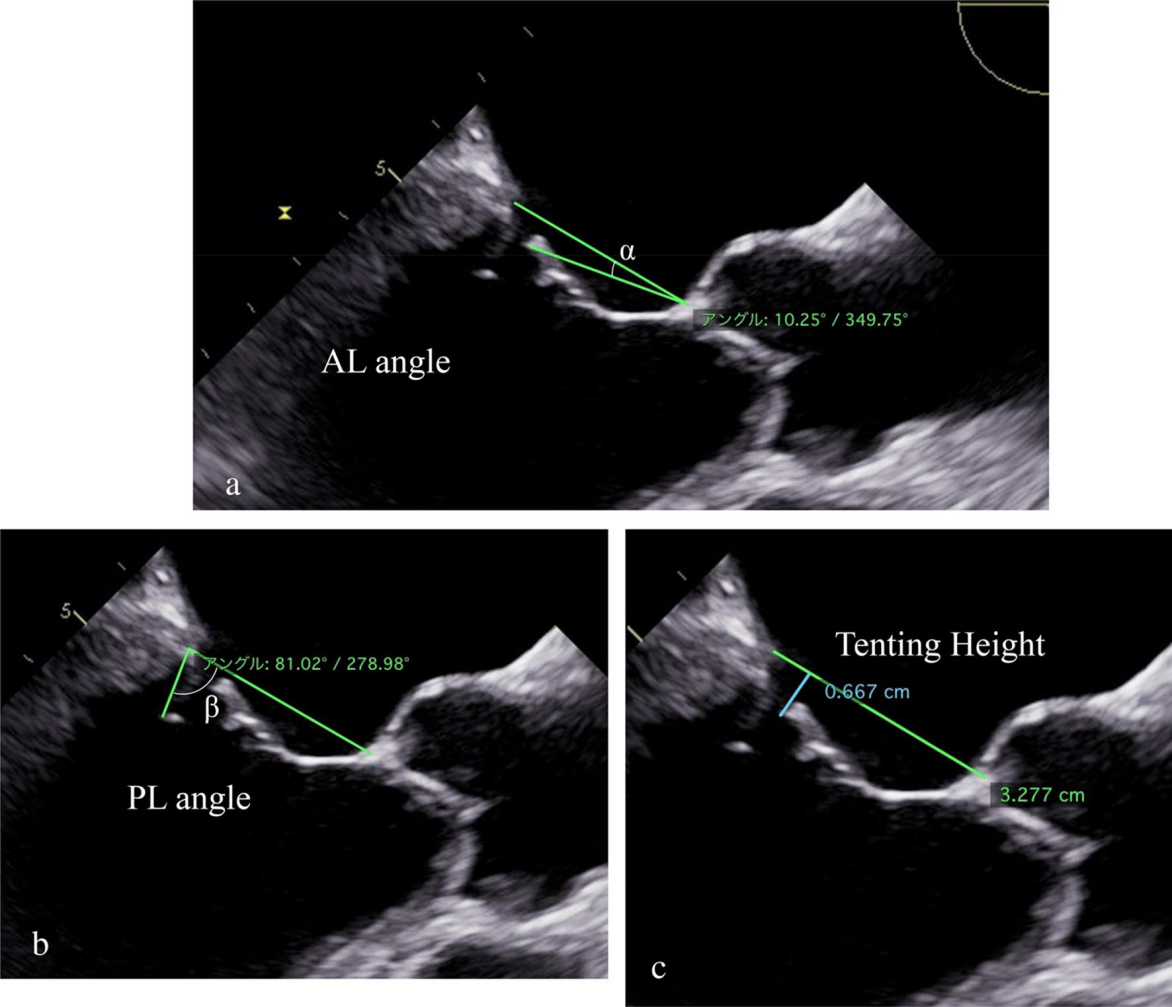
Preoperative transesophageal echocardiography at a long-axis view. **a** The α indicates the anterior mitral leaflet tethering angle (AL angle). **b** The β indicates the posterior mitral leaflet tethering angle (PL angle). **c** Tenting height was defined by the distance between the point of leaflet coaptation and the annular plane (blue line).

All patients underwent preoperative transthoracic echocardiography (TTE) (Table 2). From the parasternal long-axis window, we measured left ventricular end-diastolic dimension (LVDd), left ventricular end-systolic dimension (LVDs), and LA dimension (LAD). MR regurgitant volume (RV) and effective regurgitant orifice area (EROA) were calculated for quantification of MR. Post-operative TTE was performed 7–10 days after surgery. After 2011, in 13 of 22 patients, we also measured anterior mitral leaflet (AL) length, posterior mitral leaflet (PL) length, tenting height, AL tethering angle, and PL tethering angle [8.9], with preoperative TEE (Fig. 2, Table 3).

**Table 2:** Preoperative TTE data in 22 patients

Variables	Total (*N* = 22)	LAP + (*N* = 9)	LAP − (*N* = 13)	*P* value (LAP + vs LAP − )
LVDd (mm)	50.1 ± 6.1	54.7 ± 3.7	47.0 ± 5.6	< 0.01
LVDs (mm)	32.6 ± 3.8	34.8 ± 2.8	31.2 ± 3.9	0.02
LVEF (%)	63.8 ± 4.6	65.5 ± 4.1	62.6 ± 4.8	0.14
LAD (mm)	62.3 ± 13.5	72.8 ± 11.4	54.9 ± 9.4	< 0.01
MR severity				
RV (ml)	60.7 ± 16.3	56.7 ± 18.4	65.9 ± 12.5	0.25
EROA (cm^2^)	0.39 ± 0.09	0.35 ± 0.09	0.44 ± 0.09	0.08

**Table 3:** Preoperative TEE data in 13 patients

Variables	Total (*N* = 13)	LAP + (*N* = 8)	LAP − (*N* = 5)	*P* value (LAP + vs. LAP − )
AL angle (°)	13.9 ± 4.7	12.6 ± 5.3	16.1 ± 2.8	0.16
PL angle (°)	55.3 ± 16.9	53.4 ± 18.8	58.3 ± 14.9	0.72
AL length (cm)	6.1 ± 1.2	6.4 ± 1.4	5.7 ± 0.7	0.30
PL length (cm)	2.7 ± 0.7	2.7 ± 0.8	2.8 ± 0.5	0.72
Tenting height (cm)	0.71 ± 0.18	0.71 ± 0.23	0.71 ± 0.04	0.97

*TTE* transthoracic echocardiography, *TEE* transesophageal echocardiography

*LVDd* Left ventricular endo-diastolic dimension, *LVDs* left ventricular endo-systolic dimension, *LVEF* left ventricular ejection fraction, *LAD* left atrial dimension, *RV* regurgitant volume, *EROA* effective regurgitant orifice area, *AL* anterior mitral leaflet, *PL* posterior mitral leaflet

### Computed tomography and X-ray

**Fig. 3:**
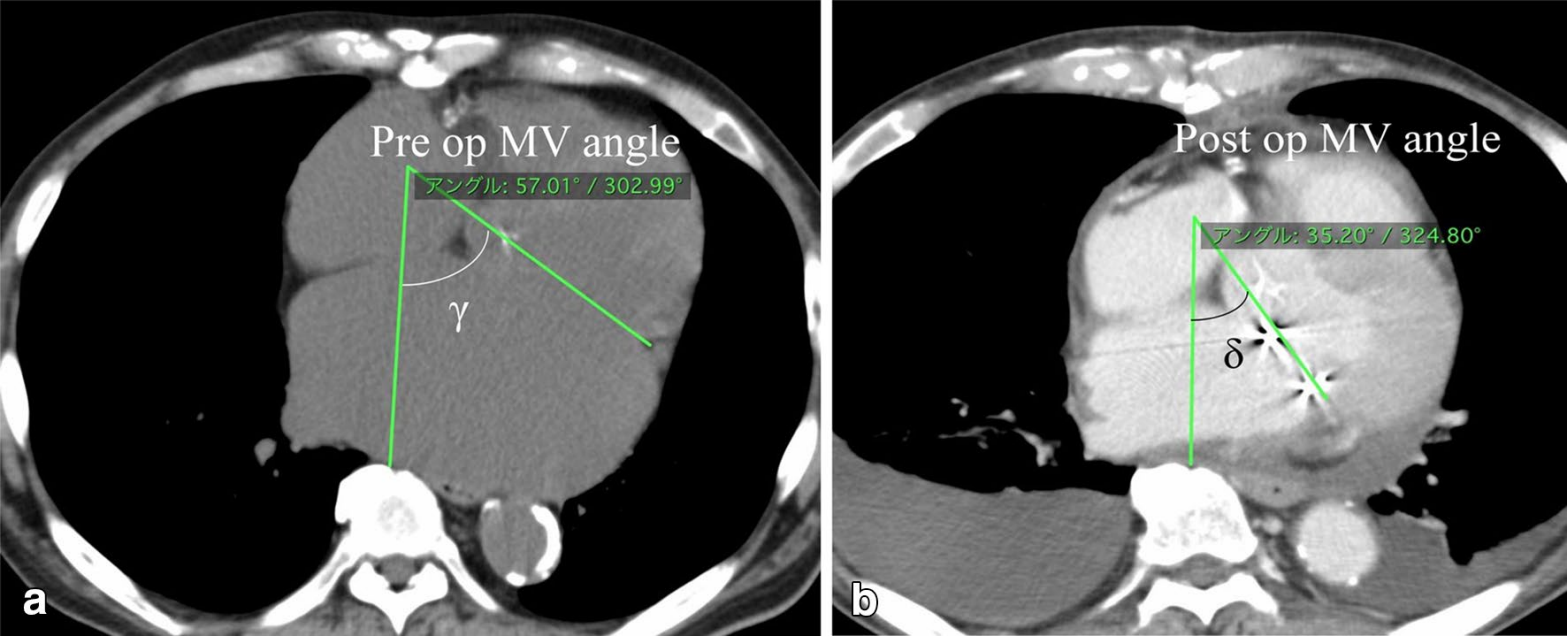
Pre and post-operative computed tomography. Mitral valve (MV) angle indicate the angle between mid-sagittal plane and the mitral annular plane. **a** The γ indicates the preoperative MV angle. **b** The δ indicates the post-operative MV angle

All patients underwent pre- and post-operative CT and X-ray. Post-operative CT and Xp data collected 7–10 days after surgery. Focusing on the AFMR-caused mitral posterior leaflet pulled backward by enlarged left atrium [12, 13], MV angle was measured by pre and post-operative CT scan (Fig. 3). MV angle indicates the angle between the mid-sagittal plane and the mitral annular plane. We compared MV angle and CTR before and after surgery.

### The surgical technique of LAP

Cardiopulmonary bypass was established using an aortic cannulation on the ascending aorta and bicaval drainage through a median sternotomy. The mitral valve was accessed via right-sided left atriotomy.

**Fig. 4:**
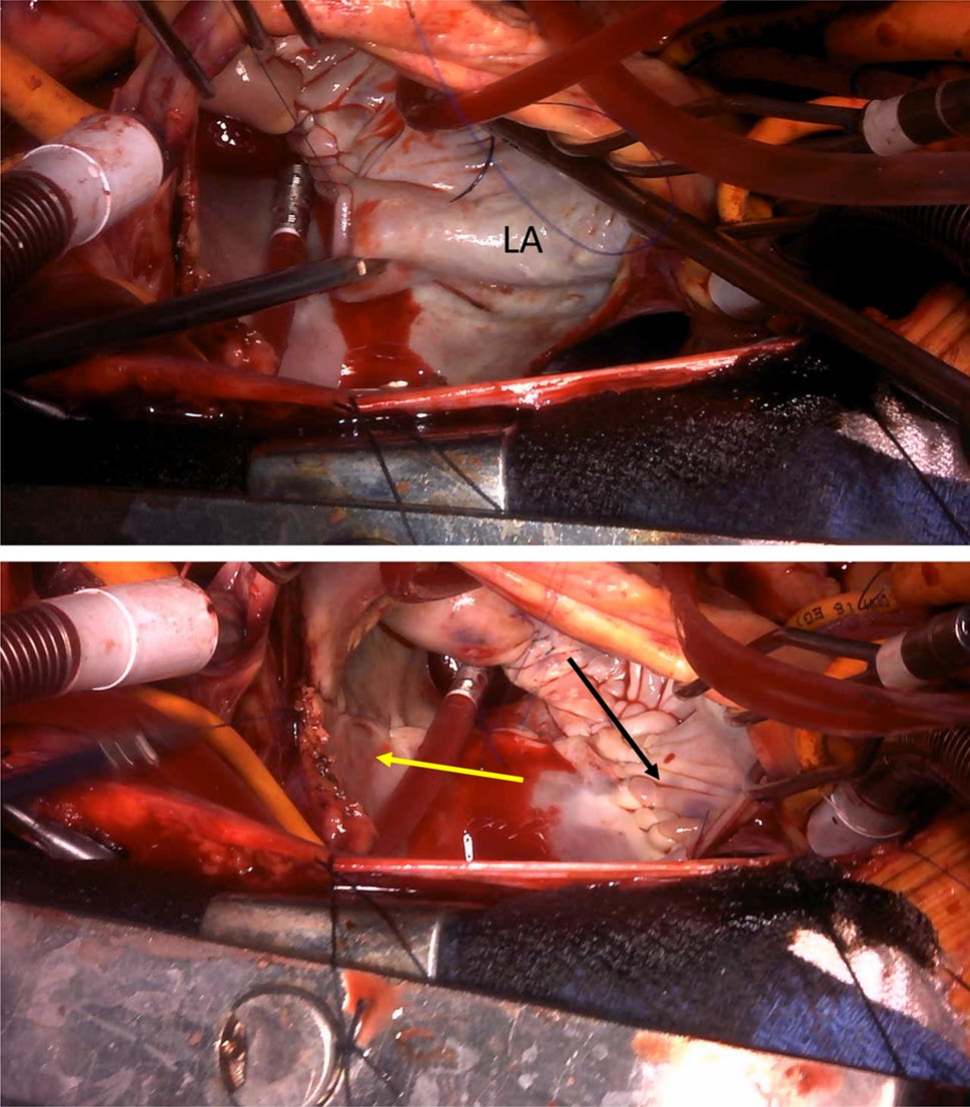
The surgical technique of left atrial plication (LAP). Black arrow indicate the LAP line between pulmonary veins and mitral valve. Yellow arrow indicates the LAP line between right pulmonary veins and left pulmonary veins

After carful observation of MV and water test, LAP performed prior to the MAP. At first, between pulmonary veins and MV, LA was plicated aggressively with a width of 4–5 cm by double layer of 4–0 polypropylene sutures from left atrial appendage to the right side of left atriotomy (Fig. 4 black arrow). Second, between left pulmonary veins and right pulmonary veins, LA posterior wall was plicated from posterior wall of LA to the left side of left atriotomy with same method described above (Fig. 4 yellow arrow). Special care must be taken to avoid injury of esophagus and tear of fragile left atrial wall.

After LAP prior to MAP, we performed water leak test. In some cases, we experienced patients whose MR controlled before doing MAP (Fig. 4).

### Statistical analysis

Categorical variables are presented as mean ± SD. Student’s *t* test was used to compare continuous variables, and chi-square test was used to compare categorical variables between the treatment groups. *P* values < 0.05 were considered statistically significant. A statistical analysis was conducted using JMP^®^ (SAS Institute Inc., Cary, NC, USA).

## Result

There were no significant differences between two groups regarding preoperative patient characteristics (Table 1). Preoperative echocardiographic data are shown in Tables 2, 3 All patients preserved LV function (EF 63.8 ± 4.6%). Preoperative LVDd (54.7 ± 3.7 vs. 47.0 ± 5.6 mm; *p* < 0.01), LVDs (34.8 ± 2.8 vs. 31.2 ± 3.9 mm; *p* = 0.02), and LAD (72.8 ± 11.4 vs. 54.9 ± 9.4 mm; *p* < 0.01) were significantly larger in the LAP + group. TEE demonstrated small PL length (2.7 ± 0.7 cm), tenting height (0.71 ± 0.18 cm), and large PL angle (55.3 ± 16.9 °°). Pre-operative RV and EROA were 60.7 ± 16.3 ml and 0.39 ± 0.09 cm^2^, respectively. Severity of MR was not significantly different between the two groups. Post MV angle demonstrated significant decrease compared with pre MV angle (40.5 ± 10.4 ° vs. 48 ± 7.9 °; *p* < 0.03).

**Fig. 5:**
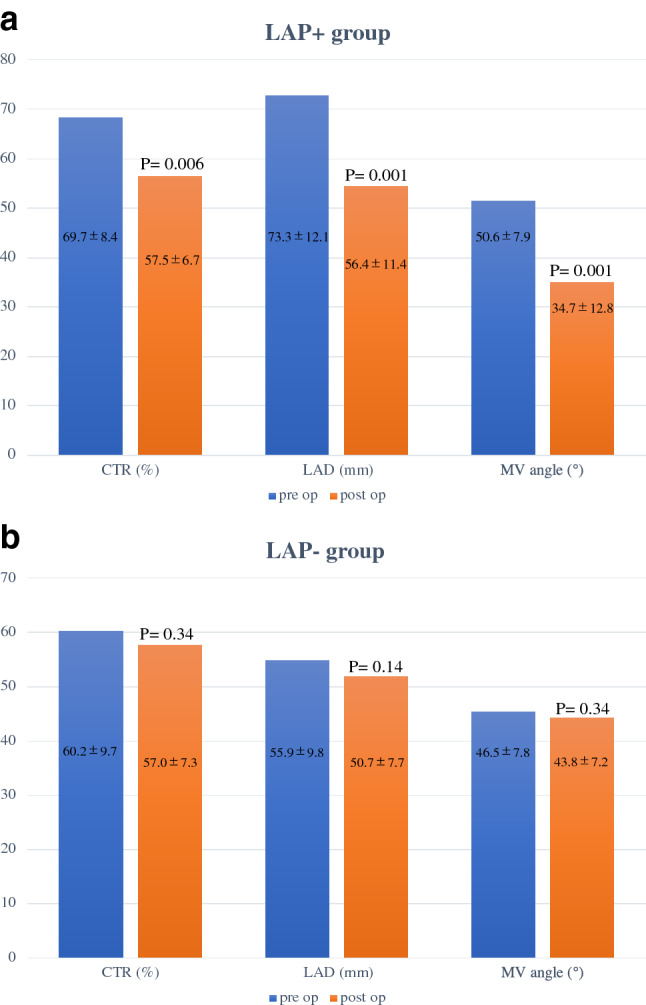
**a** Changes in cardio-thoracic ratio (CTR), mitral valve (MV) angle and left atrial dimension (LAD) before and after surgery in the LAP + group. **b** Changes in cardio-thoracic ratio (CTR), mitral valve (MV) angle and left atrial dimension (LAD) before and after surgery in the LAP − group

The LAP + group showed significant greater decrease at cardio-thoracic ratio (CTR) (11.9 ± 4.7% vs 2.7 ± 6.0%, *p* < 0.01), MV angle (16.6 ± 8.1° vs 1.2 ± 6.8°, *p* < 0.01) and LAD (18.4 ± 7.0 mm vs 6.9 ± 14.6 mm, *p* = 0.02) compared with LAP − group (Fig. 5a, b).

Patient’s perioperative profiles are presented in Table 4. Mean perfusion time was 150 ± 35 (88–232) min, and the cross-clamp time was 115 ± 26 (72–160) min. Mitral valve replacement (MVR) was performed in 6 patients and mitral ring annuloplasty (MAP) in 16 cases, respectively. In the LAP − group, additional mitral valve plasty (MVP) performed including use of artificial chordae in 3 patients and edge to edge repair in 1 patient. In contrast, in the LAP + group, we performed only LAP and MAP (*n* = 8) or MVR (*n* = 1) to correct MR. Concomitant tricuspid valve replacement (TVR) was performed in 2 patients (9.1%), tricuspid ring annuloplasty (TAP) in 17 patients (77.3%), Maze procedure in 9 patients (40.9%), coronary artery bypass grafting (CABG) in 3 patients (9.1%), and hemi- or total arch replacement (HAR or TAR) in 3 patients (9.1%). The larger size of MAP ring (30.0 ± 2.1 mm vs 27.3 ± 1.0 mm, *p* = 0.01) was attached in the LAP + group.

There were no hospital deaths. Post-operative complications that occurred were pacemaker implantation in 1 patient, reexploration for bleeding in 3 patients, and renal failure needing hemodialysis in 1 patient, and mediastinitis in 1 patient.

Duration of clinical follow-up was 3.58 ± 3.01(0.1–12.1) years. There was no re-admission for heart failure or cardiac-related death; 3 deaths occurred during the follow up period: one cerebral hemorrhage, one malignancy, and one pneumonia. No recurrence of MR or structural valve deterioration (SVD) occurred during follow-up.

## Discussion

MR associated with severe LV dysfunction due to ischemic or idiopathic myocardial disease is called functional MR (FMR). This type of MR has been widely recognized and management well debated [1–3]. Historically, Otsuji et al. showed that isolated AF with dilated mitral annulus (MA) does not cause significant MR [14]. However, recent reports revealed significant MR can occur in AF patients with dilated MA and LA [4–7]. Gentz et al. [5] reported these type of MR as “atrial functional MR (AFMR)” caused by AF and LA dilation despite preserving LV size and function. In the current era, prevalence of AFMR is reported in 4.3–7% of AF patients [5, 15]. Moreover, AFMR typically occurs in AF and heart failure with preserved ejection fraction (HFpEF). The numbers of these patients cannot be ignored. Nevertheless, current guidelines do not include management of AFMR [16, 17]. In addition, there are a few reports describing additional scope of surgical methods and results for AFMR [10, 11, 18]. While it is clear that AFMR produces good surgical outcomes, there are no reports that discuss the efficacy of left atrial plication for AFMR. This report is the first to describe the efficacy of left atrial plication for AFMR.

**Fig. 6:**
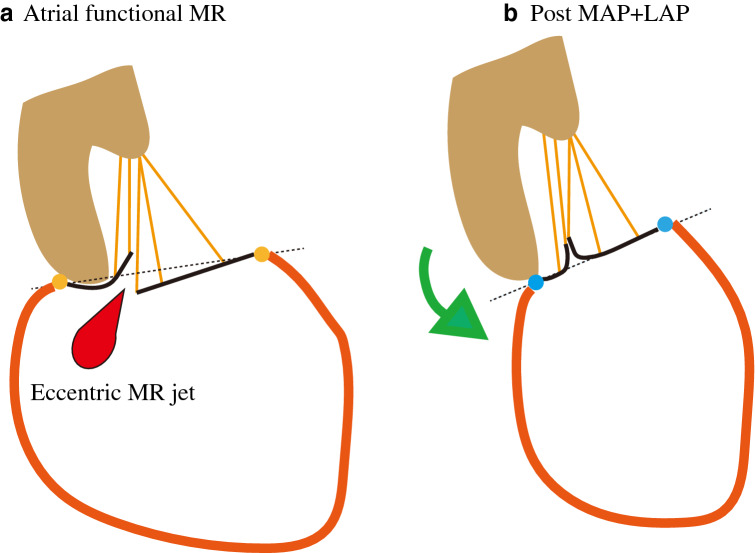
**a** In patients with AFMR, posterior annulus was displaced backward by dilated LA. As a result, PL tethering and pseudo AL prolapse occurred. The MA was dilated and horizontally inclined. **b** Post MAP and LAP for patients with AFMR. LAP resulted in correction of the horizontal inclination and pseudo AL prolapse were corrected

The mechanism of AFMR is different from “functional MR”. In AF patients, Gentz. et al. [5] showed that patients with MR had larger LA and MA compared with patients who did not have significant MR. Several reports revealed compensatory mechanism of mitral leaflet enlargement for MR, and Kagiyama et al. [19] demonstrated that patients with AFMR showed significant smaller mitral leaflets compared with the annulus (insufficient leaflet remodeling). In addition, in AFMR, LV dimension and function are preserved. As a result, mitral leaflets were flattened and tenting height relatively small compared with “functional MR” [8]. In this study, tenting height was also small (0.71 ± 0.18 cm), with no significant difference between two groups (0.71 ± 0.23 vs 0.71 ± 0.04 cm; *p* = 0.97) (Table 3). Another key mechanism of AFMR is “atriogenic leaflet tethering”. PL attaches to the junction of the LA. With LA backward enlargement, MA is displaced backward to the LA side and the PL was bent toward the LV cavity (Fig. 6). Such PL tethering is previously described as “hamstring” in rheumatic mitral stenosis [20]. In this report, PL angle is also big (55.3 ± 16.9 $^\circ$) as described in previous reports [8–10]. With extremely enlarged LA, such PL tethering and pseudo AL prolapse have been encountered in clinical practice. We surmise that correcting the horizontal inclination of a mitral valve pulled backward by the enlarged LA to perform LAP along with MAP is crucial for AFMR (Fig. 6).

Wu et al. reported that left atrial volume index is a powerful predictor for a future cardiac event [21]; Osranek et al. also reported that left atrial volume predicted adverse events in patients with lone AF [22].

In this study, LAP + group showed significant greater decrease at cardio-thoracic ratio (CTR) (11.9 ± 4.7% vs. 2.7 ± 6.0%, *p* < 0.01) and LAD (18.4 ± 7.0 mm vs. 6.9 ± 14.6 mm, *p* = 0.02) compared with LAP − group (Fig. 4, 5). Moreover, LAP + group also demonstrated significant greater decrease in MV angle (16.6 ± 8.1° vs. 1.2 ± 6.8°, *p* < 0.01) compared with LAP − group (Fig. 4, 5), which may be affect the durability of MAP. Indeed, our patients included those whose MR could be controlled after LAP before performing MAP during the surgery. In our results, although LAP + group had attachment of the larger size of MAP ring (30.0 ± 2.1 mm vs. 27.3 ± 1.0 mm, *p* = 0.01) with LV dimension larger than LAP − group (preoperative LVDd (54.7 ± 3.7 vs. 47.0 ± 5.6 mm; *p* < 0.01), LVDs (34.8 ± 2.8 vs. 31.2 ± 3.9 mm; *p* = 0.02), respectively), only 1 patient (11.1%) underwent MVR. Whereas, in the LAP − group, 5 patients (38.5%) underwent MVR. In the LAP + group, the patients did not need additional mitral valve plasty.

In our study, there were no hospital deaths, and no cardiac-related deaths nor re-admissions for heart failure during the follow-up period.

**Table 4:** Perioperative profiles

Variables	Total (*N* = 22)	LAP + (*N* = 9)	LAP − (*N* = 13)	*P* value (LAP + vs LAP − )
Perfusion time (min)	150 ± 35	144 ± 28	154 ± 40	0.49
Cross-clamp time (min)	115 ± 26	112 ± 26	118 ± 27	0.64
MVR (%)	6 (27.2)	1 (11.1)	5 (38.5)	0.14
MAP ring size (mm)	28.6 ± 2.1	30.0 ± 2.1	27.3 ± 1.0	0.01
Artificial chordae (%)	3 (13.6)	0	3 (23.1)	0.08
Edge to edge (%)	1 (4.5)	0	1 (7.7)	0.34
TVR (%)	2 (9.1)	1 (11.1)	1 (7.7)	0.80
TAP (%)	17 (77.3)	6 (66.6)	11 (84.5)	0.38
Maze (%)	9 (40.9)	2 (22.2)	7 (53.8)	0.14
CABG (%)	3 (9.1)	1 (11.1)	2 (15.4)	0.78
TAR or HAR (%)	3 (9.1)	1 (11.1)	2 (15.4)	0.78

*MVR* Mitral valve replacement, *MAP* mitral annuloplasty, *TVR* tricuspid valve replacement, *TAP* tricuspid annuloplasty, *CABG* coronary artery bypass grafting, *TAR* total arch replacement, *HAR* hemi arch replacement

In terms of the management of AFMR, Gentz et al. [5] reported that AFMR patients in continuous sinus rhythm had greater reduction of LA size and MA, and lower rate of significant MR. Therefore, they concluded AFMR may benefit from rhythm control therapy. However, most individuals who need surgery for AFMR have severely dilated LA and long-standing AF. In these patients, it is difficult to re-establish sinus rhythm. In this study, we performed Maze procedure in only nine cases (40%) and two patients recovered to sinus rhythm.

Regarding surgical procedure, Sakaguchi et al. [11] reported 12 cases of AFMR where 4 patients with LV dilation and leaflet tethering developed recurrent MR. Thus, they concluded MAP alone may not be sufficient for such patients. In our cases, LV dimension and function were preserved and there was no recurrence of MR. LAP may affect the durability of MR.

### Limitations

Limitations are as follows: this was a retrospective, single-center study. The number of enrolled patients was relatively small. Surgical procedure was decided by the individual surgeon’s preference. Finally, the follow-up period was relatively short.

## Conclusions

The results of surgery for AFMR were satisfactory. In the pathophysiology of AFMR, posterior leaflet tethering has occurred due to left atrial enlargement. It is suggested that it may be possible to correct the angle of the mitral valve tilted horizontally by LAP. More cases need to be considered in the future.
